# Transient Treg depletion enhances therapeutic anti‐cancer vaccination

**DOI:** 10.1002/iid3.136

**Published:** 2016-11-21

**Authors:** Scott A. Fisher, Wayne J. Aston, Jonathan Chee, Andrea Khong, Amanda L. Cleaver, Jessica N. Solin, Shaokang Ma, W. Joost Lesterhuis, Ian Dick, Robert A. Holt, Jenette Creaney, Louis Boon, Bruce Robinson, Richard A. Lake

**Affiliations:** ^1^ School of Medicine and PharmacologyUniversity of Western Australia, QEII Medical CentreNedlandsWestern AustraliaAustralia; ^2^ National Research Centre for Asbestos Related DiseasesQEII Medical CentreNedlandsWestern AustraliaAustralia; ^3^ British Columbia Cancer AgencyVancouverBritish ColumbiaCanada; ^4^ BiocerosUtrechtThe Netherlands

**Keywords:** Cancer immunotherapy, depletion, regulatory T cell, tumor immunology, vaccination

## Abstract

**Introduction:**

Regulatory T cells (Treg) play an important role in suppressing anti‐ immunity and their depletion has been linked to improved outcomes. To better understand the role of Treg in limiting the efficacy of anti‐cancer immunity, we used a Diphtheria toxin (DTX) transgenic mouse model to specifically target and deplete Treg.

**Methods:**

Tumor bearing BALB/c FoxP3.dtr transgenic mice were subjected to different treatment protocols, with or without Treg depletion and tumor growth and survival monitored.

**Results:**

DTX specifically depleted Treg in a transient, dose‐dependent manner. Treg depletion correlated with delayed tumor growth, increased effector T cell (Teff) activation, and enhanced survival in a range of solid tumors. Tumor regression was dependent on Teffs as depletion of both CD4 and CD8 T cells completely abrogated any survival benefit. Severe morbidity following Treg depletion was only observed, when consecutive doses of DTX were given during peak CD8 T cell activation, demonstrating that Treg can be depleted on multiple occasions, but only when CD8 T cell activation has returned to base line levels. Finally, we show that even minimal Treg depletion is sufficient to significantly improve the efficacy of tumor‐peptide vaccination.

**Conclusions:**

BALB/c.FoxP3.dtr mice are an ideal model to investigate the full therapeutic potential of Treg depletion to boost anti‐tumor immunity. DTX‐mediated Treg depletion is transient, dose‐dependent, and leads to strong anti‐tumor immunity and complete tumor regression at high doses, while enhancing the efficacy of tumor‐specific vaccination at low doses. Together this data highlight the importance of Treg manipulation as a useful strategy for enhancing current and future cancer immunotherapies.

## Introduction

Regulatory T cells (Treg) are a subset of CD4 lymphocytes with immunosuppressive activity characterized by the expression of the transcription factor forkhead box protein 3 (FoxP3) 1, 2. Treg play an essential role in preventing chronic inflammatory, allergic, and autoimmune diseases by regulating and maintaining immune homeostasis. The importance of Treg to immune homeostasis is underpinned by the fact that dysfunctional FoxP3 expression is associated with severe, often fatal, lymphoproliferative disease as observed in the scurfy mouse phenotype 3, 4 and human IPEX (immune dysregulation, polyendorinopathy, enteropathy, X‐linked) syndrome 5, 6. Treg can be classified into two distinct types based on their ontogeny 7. Thymic Treg (tTreg) develop in the thymus and constitutively express CD4, CD25, CTLA‐4, GITR, and FoxP3 before entering the circulation where, they function to maintain peripheral tolerance and limit autoimmunity via direct interaction with dendritic cells (DCs) and/or effector T cells (Teff) 2, 8, 9. Conversely, peripheral Treg (pTreg) are derived from naïve (CD4+CD25‐FoxP3‐) peripheral CD4 T cells in which, FoxP3 expression is induced via TGF‐β 10, 11, 12 allowing pTreg to regulate local immune pathologies in tissues in a cell contact independent, cytokine dependent manner 9.

The role of Treg in maintaining immune homeostasis and peripheral tolerance is crucial in the context of preventing allergy and autoimmunity. However, as many tumor antigens are either overexpressed or mutated self‐antigens, Treg induced immune homeostasis can significantly limit the efficacy of anti‐tumor immunity. Early studies demonstrated that depletion of CD4+CD25+ Treg with antibodies, targeting the IL‐2 receptor (CD25) restored anti‐tumor immunity in a variety of pre‐clinical models 13, 14, 15, 16, 17, 18, 19. While, these studies highlighted the therapeutic potential of Treg depletion, anti‐CD25 therapy is not Treg‐specific as it also depletes CD25+ effector T cells, the very cells that drive anti‐tumor immunity, thus restricting the efficacy of anti‐CD25 therapy to a prophylactic setting 16, 20, 21, 22.

To overcome this issue, transgenic mice were created in which the Diphtheria toxin receptor (dtr) is expressed as a fusion protein with green fluorescent protein (GFP) under the control of the *FoxP3* promoter. C57Bl/6 DEREG (DEpletion of REGulatory T cells) 23 and BALB/c FoxP3.dtr 24 mice allow selective depletion of Treg following administration of Diphtheria toxin (DTX), without affecting other effector T cell subsets. Numerous studies using DEREG mice have shown the potential of targeted Treg depletion to augment pathological and therapeutic outcomes in autoimmune 23, 24, infectious diseases 25, 26 and cancer settings 27, 28, 29, although some limitations have been identified with this model 25.

Given the recent advances in the use of immune based cancer therapies, particularly for solid cancers 30, 31, the role of Treg suppression in limiting anti‐tumor immunity is of great interest. Technological innovations in next generation sequencing (NGS) have enabled the identification of tumor‐specific mutated antigens (i.e., neo‐antigens) that are uniquely recognized by the host's immune system 32, 33, 34, 35, 36. The ability to identify immunogenic tumor neo‐antigens brings cancer immunotherapy to a position where, the development of a patient‐specific anti‐cancer vaccine is now a reality 35, 37, 38. The combination of novel therapeutic approaches, such as Treg depletion, that selectively enhance host immunity to tumor neo‐antigens will be a major driving force behind the future success of cancer immunotherapy.

Here, we demonstrate the use of the BALB/c FoxP3.dtr transgenic mouse, as an ideal alternative model with which to demonstrate the full therapeutic potential of Treg depletion to boost the efficacy of host anti‐tumor immunity. We show that DTX‐mediated Treg depletion is transient and dose‐dependent; leading to strong anti‐tumor immunity and complete tumor regression at high doses, while enhancing the efficacy of tumor‐specific vaccine immunotherapy at low doses.

## Results

### DTX‐mediated regulatory T cell depletion occurs in a transient, dose‐dependent manner

**Figure 1:**
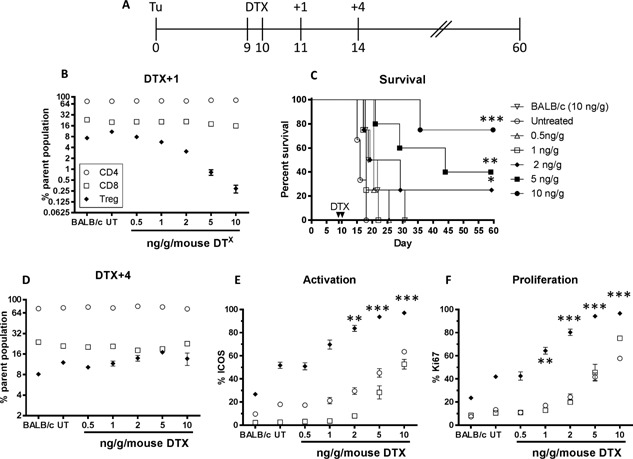
Transient dose‐dependent Treg depletion. AB1‐HA bearing BALB/c FoxP3.dtr mice treated with indicated doses of DTX on days 9 and 10. Treg‐specific depletion in peripheral blood shown 1 (DTX+1) and 4 (DTX+4) days after DTX administration. (A) Experimental design. (B) DTX dose‐dependent depletion of Treg at DTX+1. (C) Survival outcome. (D) Rebound of T cell subsets and (E) T cell activation (ICOS) and (F) proliferation (Ki67) status four days after transient DTX‐mediated Treg depletion. Representative data from three independent experiments showing mean (*n* = 5/group) ± SEM. CD4 (open circle) and CD8 (open square) as proportion of total lymphocytes. Treg (closed diamond) as proportion of total CD4 T cells. Log rank (Mantel–Cox) analysis was performed on survival curves. Mann–Whitney U‐test for comparisons between two groups. *P < 0.05, **P < 0.01, ***P < 0.001. UT, untreated; Tu, (AB1‐HA) tumor cells.

To determine the efficacy of DTX‐mediated Treg depletion in tumor bearing BALB/c FoxP3.dtr mice, we administered DTX on two consecutive days and assessed Treg number in peripheral blood by flow cytometry (Fig. 1). DTX‐mediated Treg depletion occurred in a dose‐dependent manner with maximum depletion observed on the day following the last DTX dose (DTX+1), with a significant decrease (*P* < 0.01) in Treg observed for DTX doses greater than 1 ng/g compared to PBS (untreated; UT) controls. Importantly, DTX treatment was Treg‐specific as it did not affect non‐Treg lymphocyte populations (i.e. CD4 or CD8) in BALB/c FoxP3.dtr treated mice, nor did DTX treatment deplete Treg in, or have any effect on lymphocytes numbers in wildtype BALB/c mice, even at the highest dose (Fig. 1B).

DTX‐mediated Treg depletion was transient, with the number of Treg rebounding to baseline levels by 4 days post treatment (DTX+4, Fig. 1D). Compared to controls, Treg rebound was associated with a significant increase in expression of the surface markers ICOS and Ki67, indicating increased Treg activation and proliferation respectively. Interestingly, while there was no significant change to the number of CD4 and CD8 T cells, transient depletion and rebound of Treg correlated with a significant increase (*P* < 0.01) in total CD4 and CD8 T cell activation and proliferation, particularly at DTX doses above 2 ng/g (Fig. 1E and F). Despite the transient nature of DTX‐mediated Treg depletion, a significant survival benefit was observed for mice receiving DTX doses above 2 ng/g (Fig. 1C).

### Limited Treg repopulation and CD8 T cell activation in tumors following DTX treatment

**Figure 2:**
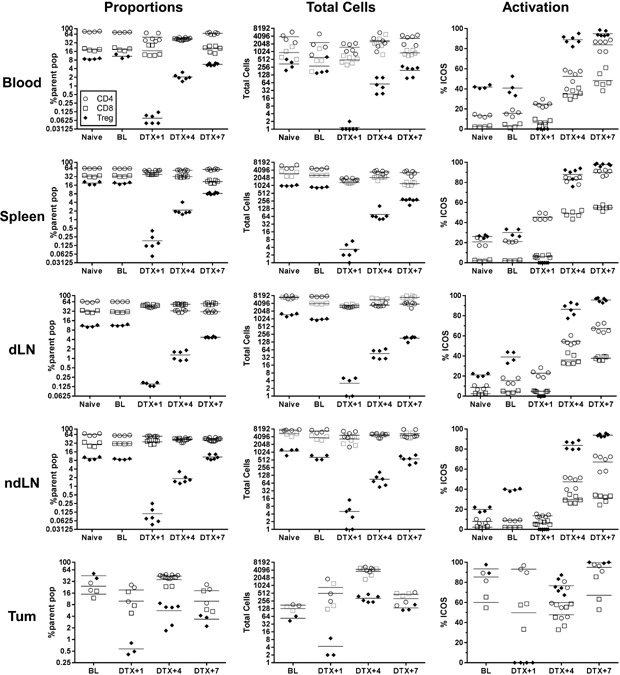
Systemic Treg depletion following DTX treatment. AB1‐HA bearing mice treated with 25 ng/g/mouse i.p. of DTX on days 9+10 post tumor inoculation. Figure shows the kinetics of Treg‐specific depletion and subsequent Treg (closed diamond), CD4 (open circle) and CD8 (open square) T cell activation status (ICOS expression) in peripheral blood, spleen, draining lymph node (dLN), non‐draining lymph node (ndLN) and tumor at baseline (BL) and 1 (DTX+1), 4 (DTX+4) and 7 (DTX+7) days after DTX administration. Pooled data shown for individual mice from two independent experiments. CD4 and CD8 as proportion of total lymphocytes (100,000 events collected). Treg as proportion of total CD4 T cells. Bars represent mean (*n* = 2–6/group). Mann–Whitney U‐test for comparisons between two groups.

We next sought to determine whether the effect of DTX‐mediated Treg depletion in peripheral blood was representative of Treg depletion in other organs. In this experiment, tumor bearing BALB/c FoxP3.dtr mice were treated with 25 ng/g of DTX on two consecutive days and Treg depletion in the blood was compared to spleen, tumor, draining and non‐draining lymph nodes using flow cytometry (Fig. 2). Maximum Treg depletion was observed in all compartments with less than 1% Treg remaining at DTX+1. As with the previous experiment, DTX‐mediated Treg depletion was transient, with Treg numbers normalized by DTX+4 and returning to near baseline levels in the blood, spleen and lymph nodes by DTX+7. Again, Treg depletion and recovery correlated with a significant increase (*P* < 0.01) in the activation status (ICOS expression) on all T cell populations at DTX+4 and DTX+7. A similar increase in T cell proliferation status was also observed (Ki67 expression, data not shown). Interestingly, the kinetics of Treg recovery in the tumor was less robust compared to the other organs and the level of CD8 T cell activation not as pronounced, possibly reflecting the relatively high level of T cell activation already within the tumor prior to Treg depletion.

### Timing of consecutive Treg depletion is important to avoid morbidity

**Figure 3:**
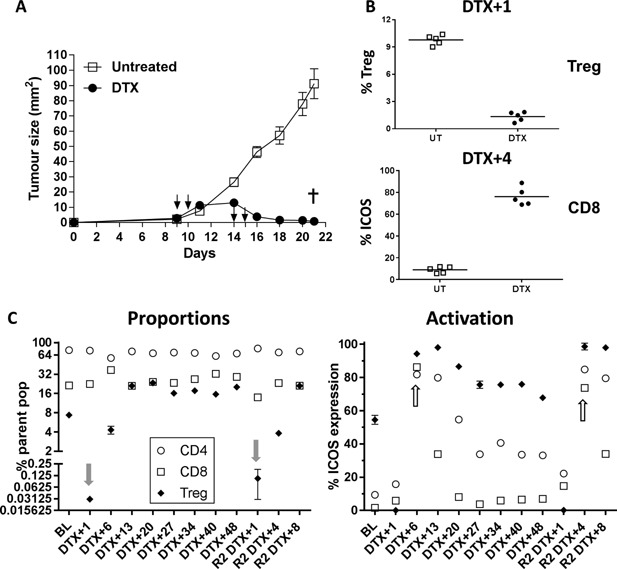
Repeat Treg depletion. Repeat rounds of DTX medicated Treg depletion in BALB/c FoxP3.dtr mice treated with 25 ng/g/mouse DTX i.p. (A) AB1‐HA tumor growth in BALB/c FoxP3.dtr mice treated with two rounds of DTX (black arrows) 1day apart. (B) Transient Treg depletion in blood correlated with increase CD8 T cell activation in DTX‐treated mice. (C) Changes in CD4, CD8, and Treg proportions and activation status (ICOS expression, *P* < 0.01) in peripheral blood over time. T cell activation (white arrows) correlated with transient Treg depletion (grey arrows). All mice remained healthy after receiving second round of the same dose of DTX (R2). Representative data showing mean (*n* = 5/group) ± SEM from two independent experiments. CD4 (open circle) and CD8 (open square) as proportion of total lymphocytes. Treg (closed diamond) as proportion of total CD4 T cells. Mixed model analysis of variance was used to analyse difference in growth. Mann–Whitney U‐test two groups *P < 0.05, **P < 0.01, ***P < 0.001.

For BALB/c FoxP3.dtr mice to be a useful model for assessing the influence of Treg during tumor development, we investigated whether Treg could be depleted on multiple occasions. Tumor bearing BALB/c FoxP3.dtr mice were treated with two rounds of DTX given 4 days apart (i.e., R1 days 9 + 10 and R2 days 14 + 15) or left untreated (Fig. 3A). As expected, transient Treg depletion was associated with increased CD8 T cell activation and correlated with complete tumor regression compared to untreated mice (Fig. 3A and B). However, despite this positive response, all DTX‐treated mice became severely moribund after the second round of DTX treatment and were euthanized. We hypothesized that the severe morbidity associated with the second DTX treatment occurred at the peak of CD8 T cell activation. To test this hypothesis, BALB/c FoxP3.dtr mice were treated with 25 ng/g DTX and the level of Treg depletion/recovery and subsequent CD8 T cell activation over multiple time points was assessed by flow cytometry (Fig. 3C). As with previous experiments, Treg depletion was transient with maximum Treg depletion occurring at DTX+1 and Treg rebound was evident by DTX+6. The rebound of Treg was maintained at two‐fold higher than baseline at DTX+13 and remained at this level until the second round of DTX was given at DTX+48; at which point the cycle of DTX‐mediated Treg depletion and rebound was repeated. As expected, Treg depletion and rebound correlated with increased T cell activation, with peak CD8 T cell activation occurring at DTX+6 (>80% ICOS expression, *P* < 0.001) followed by a decline to baseline levels by DTX+20. Interestingly, the kinetics of Treg and CD4 activation mirrored that of CD8 T cells, although CD4 activation never returned to baseline levels, instead remaining elevated until the second round of DTX. Throughout the duration of this experiment (100 days total) all mice remained healthy, suggesting that a second dose of DTX can be given, without major side effects, but only after CD8 T cell activation has returned to baseline.

### CD8 T Cells are necessary but not sufficient for effective anti‐tumor immunity after Treg depletion

**Figure 4:**
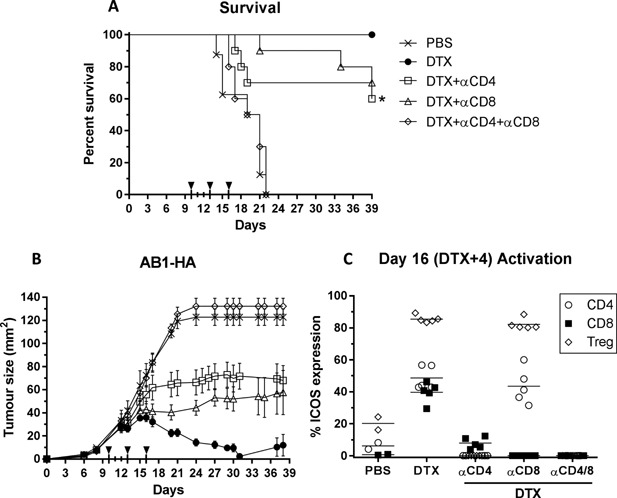
Effector T cells drive anti‐tumor response in the absence of Treg. (A) Survival and (B) tumor growth curves and (C) peak T cell activation (DTX + 4) in peripheral blood of tumor bearing mice following Treg depletion in the presence or absence of effector T cells. AB1‐HA tumors were inoculated on day 0 and mice treated with 10 ng/g/mouse DTX on days 11 and 12. Depleting antibodies were administered i.p. on days 10, 13 and 16. Data are mean (*n* = 8–10/group) ± SEM from two independent experiments. Log rank (Mantel–Cox) analysis was performed on survival curves. Mixed model analysis of variance was used to analyse difference in growth. Mann–Whitney U‐test *P < 0.05.

Antigen‐specific CD8 T cells are a crucial component of an effective anti‐tumor immune response. To assess the role of CD8 T cells in driving anti‐tumor immunity in the absence of Treg, tumor bearing BALB/c FoxP3.dtr mice were left untreated (PBS), or treated with CD4 or CD8 depleting antibodies, alone or in combination, in the context of DTX‐mediated Treg depletion (Fig. 4). Consistent with our previous experiments, we observed a significant survival benefit in DTX‐treated mice compared with PBS control mice. Depletion of both CD4 and CD8 T cells (open diamonds) completely abrogated any survival benefit associated with DTX‐mediated Treg depletion, confirming that Teffs are necessary for the response (Fig. 4A). A significant delay (*P* < 0.01) in tumor growth, but not complete tumor regression, was observed following depletion of either CD4 or CD8 T cells. Despite effective Treg depletion in all DTX treatment groups (data not shown), the increase in CD8 T cell activation was almost five‐fold lower in CD4 depleted mice compared to the DTX only group (7.9% vs. 39% ICOS expression, respectively), while the level of CD4 activation remained unchanged in CD8 depleted mice (Fig. 4C). Taken together, these data suggest a requirement for CD4 ‘help’ in the generation of an effective CD8 anti‐tumor immunity. However, tumor regression in the absence of CD8 T cells suggests that while, CD8 T cells are necessary, they are not sufficient for compete tumor regression observed in the absence of Treg.

### The efficacy of Treg depletion is diminished with increased tumor burden

**Figure 5:**
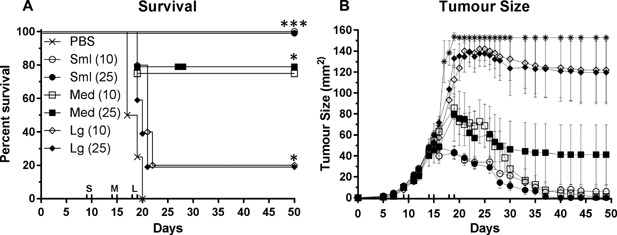
The efficacy of Treg depletion is diminished with increased tumor burden. Mice bearing small (circles; ≤25 mm^2^), medium (squares; 40 mm^2^ <60 mm^2^) or large (diamonds; >90 mm^2^) AB1‐HA tumors were treated with either 10 ng/g or 25 ng/g DTX (Black arrows; S: D9/10, M: D14/15 and L: D18/19) and tumor growth and survival monitored. (A) Overall survival and (B) tumor growth curves demonstrating that Treg depletion is most effective on small tumors and is diminished as tumor burden increases. tumor growth data are mean (*n* = 5/group) ± SEM. Log rank (Mantel–Cox) analysis was performed on survival curves. Mixed model analysis of variance was used to analyse difference in growth. *P < 0.05, ***P < 0.001.

So far we have demonstrated that DTX‐mediated Treg depletion in tumor bearing BALB/c FoxP3.dtr mice is associated with significant increases in CD8 and CD4 T cell activation and an overall survival benefit, compared to untreated controls. In all experiments, DTX was administered on two consecutive days, when tumors were relatively small (∼25–30 mm^2^). To assess whether tumor burden influenced the efficacy of DTX‐mediated anti‐tumor immunity, we administered DTX to BALB/c FoxP3.dtr mice bearing small (≤25 mm^2^, days 9 + 10), medium (40 mm^2^ <60 mm^2^ days 14 + 15), or large (>90 mm^2^, days 18 + 19) tumors and assessed tumor growth (Fig. 5). Consistent with previous experiments, complete tumor regression and a significant survival benefit was observed in mice bearing small tumors at both doses of DTX. A similar outcome was observed for mice bearing medium sized tumors with 80% of these mice showing almost complete tumor regression and significantly improved overall survival compared to UT controls. Conversely, only 20% of mice bearing large tumors demonstrated a complete response to Treg depletion.

### Depletion of Treg as an effective immunotherapy against different tumor cell lines

**Figure 6:**
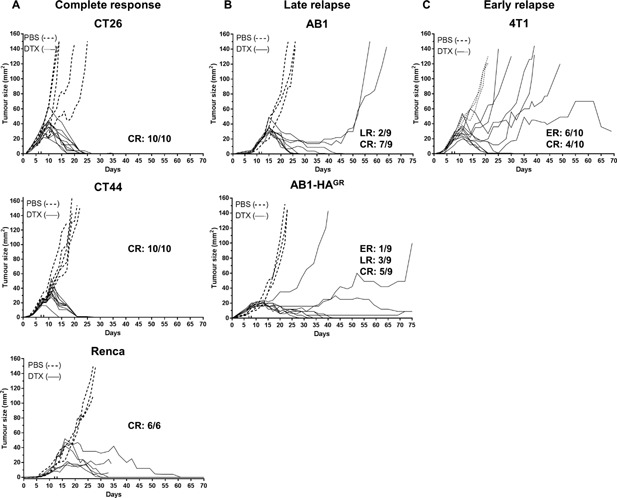
Treg depletion is effective against different tumor cell lines. BALB/c FoxP3.dtr mice inoculated with indicated tumor cell lines were treated with PBS (dashed lines) or 10 ng/g/mouse DTX (solid lines) at indicated time points (small upward dashes on X axis) and tumor growth assessed. All tumors responded to Treg depletion, but the overall response varied between (A) complete response with no relapse to partial anti‐tumor immunity with either (B) late relapse, or (C) early relapse. CR, complete response; ER, early relapse; LR, late relapse. Each line represents one mouse. Combined data from two independent experiments (*n* = 6–10 mice/group) with each line representing growth of an individual tumor. Data are from two independent experiments. Mixed model analysis of variance was used to analyse difference in growth.

To establish the use of BALB/c FoxP3.dtr mice as a suitable model to investigate the influence of Treg during tumor development we assessed the capacity of Treg depletion to induce anti‐tumor immunity against a variety of different tumor cell lines. Mice were inoculated with either mesothelioma (AB1 and AB1‐HA^GR^), renal cell carcinoma (Renca), colon carcinomas (CT26 and CT44), or mammary carcinoma (4T1) cell lines and 10 ng/g DTX given when tumors were ∼20 mm^2^. Consistent with previous experiments, Treg depletion was transient and induced strong CD8 T cell activation (data not shown) and was associated with significant tumor regression (*P* < 0.001) in tumor bearing mice compared to respective PBS controls (dotted lines, Fig. 6). Interestingly, the kinetics of tumor regression varied between different tumor types with the renal and colon carcinomas showing a ‘complete response’ without any relapse in 100% of DTX‐treated mice. As previously shown, mice bearing AB1‐HA related mesothelioma tumors all responded to DTX treatment, although a late relapse was observed in a small number of the parental AB1 (*n* = 2) and AB1‐HA^GR^ (*n* = 4) bearing mice. A single AB1‐HA^GR^ tumor bearing mouse displayed an early relapse (Fig. 6B). Conversely, the mammary carcinoma cell line 4T1 displayed a mixed, but significant response to Treg depletion (Fig. 6C), ranging from complete regression in 40% of mice to partial response, followed by early relapse in the remaining 60% DTX‐treated mice. Importantly, all complete responders, regardless of tumor type, remained tumor free for the duration of the experiment (at least 69 days), while those who relapsed early failed to control tumor growth. Late tumor relapse varied between tumor outgrowth and stable disease.

### Minimal Treg depletion enhances efficacy of peptide vaccine immunotherapy

**Figure 7:**
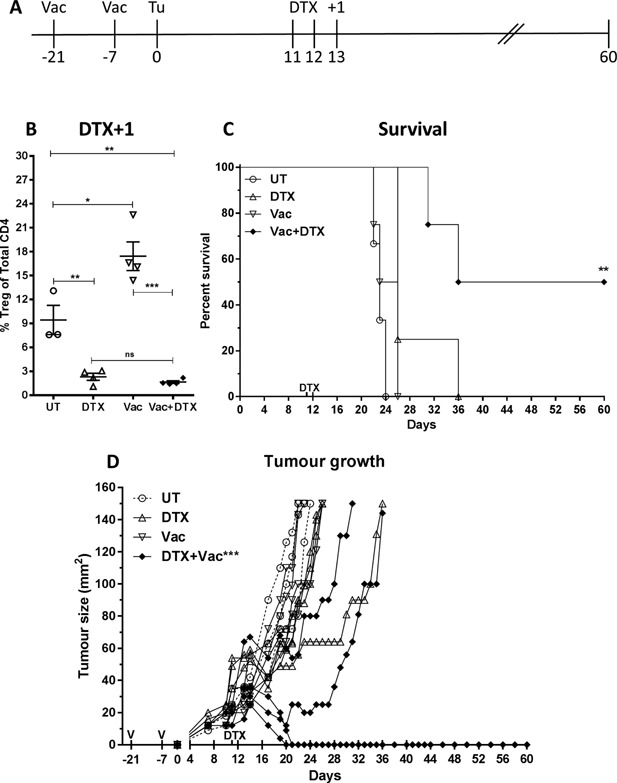
Treg depletion improves peptide vaccine efficacy. Four groups of BALB/c FoxP3.dtr mice were inoculated with AB1‐HA tumor cells (day 0) and were left either (1) untreated; (2) received the CL4 peptide at ‐21 and ‐7 days prior to tumor inoculation; (3) treated with 2 ng/g/mouse DTX on days 11 and 12 or (4) received both vaccine plus DTX. (A) Experimental design. (B) Treg as a proportion of Total CD4 T cells one day after DTX administration in peripheral blood. Data (*n* = 4/group) ± SEM. (C) Overall survival and (D) individual tumor growth curves demonstrating significant improvement in vaccine efficacy in combination with minimal transient Treg depletion. Log rank (Mantel–Cox) analysis was performed on survival curves. Mixed model analysis of variance was used to analyse difference in growth. Mann–Whitney U‐test between two groups. *P < 0.05. *P < 0.05, **P < 0.01, ***P < 0.001.

DTX‐mediated, transient Treg depletion at doses ≥10 ng/g is sufficient to induce a strong anti‐tumor immunity leading to eradication of tumors in BALB/c FoxP3.dtr mice. While, acute Treg‐specific depletion is not currently possible as an immunotherapy for most human cancers, we hypothesized that minimal, depletion of Treg might improve the efficacy of a tumor‐specific peptide vaccine. To test this hypothesis, four groups of BALB/c FoxP3.dtr mice were inoculated with AB1‐HA tumor cells on day zero and were either left (1) untreated; (2) received the HA MHC class I immunodominant epitope ‘CL4’ as peptide vaccination only on days ‐21 and ‐7 prior to tumor inoculation, (3) given 2 ng/g DTX only on days 11 and 12 or (4) received both peptide vaccine plus DTX (Fig. 7A). As previously shown (Fig. 1B), treatment with a lower dose of DTX‐reduced Treg relative to UT controls by approximately 30% (*P* < 0.01, 2–3% vs. 9% respectively). Conversely, vaccination alone induced a significant increase in Treg compared to untreated controls (*P* < 0.05, 17% vs. 9% respectively, Fig. 7B). Neither peptide vaccination nor minimal Treg depletion alone could prevent tumor outgrowth. However, combining vaccination and minimal Treg depletion led to a significant delay in tumor growth (*P* < 0.001) and improved overall survival (*P* < 0.01), with complete tumor regression observed in half of treated mice compared to all other groups (Fig. 7C and D). Taken together, these data indicated that even a minimal, temporary decrease in Treg is sufficient to significantly improve the efficacy peptide vaccination immunotherapy.

### Discussion and Conclusions

Since, the discovery of Treg as a key player in orchestrating immune homeostasis, the potential for their use as a therapeutic tool to augment immune tolerance has garnered considerable interest. This is particularly true for the field of cancer immunotherapy, where Treg suppression can potentially override the development of an effective anti‐cancer immune response. In this study, we used the BALB/c FoxP3.dtr transgenic mouse model to investigate the role of Treg in limiting the efficacy of anti‐cancer immunity and immunotherapy. We chose this model because unlike in other Treg depletion studies, DTX‐mediated depletion is Treg‐specific and represents an efficient way to decrease Treg number without affecting effector T cell populations.

We found that DTX‐mediated Treg depletion was a transient, dynamic process that occurred in a dose‐dependent manner. Maximum Treg depletion followed by recovery occurred at 1 (DTX+1) and 4–6 (DTX+4–6) days after DTX treatment, respectively. Consistent with a previous study in which BALB/c FoxP3.dtr mice were used to access the impact of Treg depletion in an autoimmune model of gastritis 39, Treg recovery was associated with effector T cell activation and proliferation. While we did not directly assess cytokine production, Nyström et al., 39 demonstrated increased expression of TNFα and IFNγ in lymphnode and spleens from DTX‐treated BALB/c FoxP3.dtr mice, an outcome that was consistent with the significant delays and often complete regression in tumor growth and associated survival benefits observed for all tumor lines we tested. Similarly, we observed persistent activation of CD4 T cells after DTX treatment. However, in contrast to Nyström and other studies using the C57Bl/6 DEREG model 23, 24, 25, any signs of potential DTX related morbidity were only observed when Treg depletion corresponded with peak CD8 T cell activation. While, morbidity in the studies using C57Bl/6 DEREG mice could be associated with their use of slightly higher doses of DTX (1–3 times higher than our study), this is unlikely to be the case with BALB/c FoxP3.dtr mice as the Nystrom study used an equivalent doses to us. Taken together, our data suggest that a second round of Treg depletion can be given, without major side effects once CD8 T cell activation has returned to baseline levels; making the BALB/c FoxP3.dtr model ideal for longitudinal studies.

The crucial role of tumor‐specific effector T cells in the development of effective anti‐tumor immunity was confirmed by depletion studies. Consistent with other studies 27, 28, 40, combined depletion of CD8 and CD4 subsets completely abrogated any tumor regression or survival benefit associated with Treg depletion. Surprisingly, and in addition to the requirement of CD4 help in achieving maximum CD8 T cell activation, we observed tumor regression in the absence of CD8 T cells. A similar outcome has been reported in the DEREG model 28, which was subsequently shown to be associated with increased natural killer (NK) cell activity following Treg depletion. These data suggest that Treg may control multiple elements at the nexus of innate and adaptive immune responses that ultimately impact on the development of effective anti‐tumor immunity.

Finally, we used the BALB/c FoxP3.dtr transgenic mouse model to confirm therapeutic potential of Treg‐specific depletion to significantly enhance anti‐tumor immunity when used in combination with peptide vaccine immunotherapy. We found that minimal Treg depletion combined with peptide vaccination correlated with significant tumor regression and survival. These data are consistent with other pre‐clinical studies in which selective depletion of Treg in DEREG mice improved the efficacy of therapeutic vaccination against B16 melanoma 27, 28. Given the recent advances in the identification of tumor neo‐antigens 32, 33, 34, 35, 36, and progress toward the development of patient‐specific cancer vaccines 37, 38, 41, our findings indicate that targeted modulation of Treg would synergise favorably with other cancer immunotherapies.

In conclusion, we show that the BALB/c FoxP3.dtr mouse model is an ideal alternative model with which to study the influence of Treg in modulating the development of an effective anti‐tumor immune response, without any of the complications observed in the DEREG model 25 and highlights the importance of developing strategies that incorporate the targeting of Treg in the development of future cancer immunotherapies.

## Materials and Methods

### Mice

BALB/C (H‐2 K^d^) wild type and BALB/c FoxP3.dtr.crlsc expressing the Diphtheria toxin receptor (dtr) under control of the FoxP3 promoter 24 mice aged between 6 and 12 weeks were obtained from the Animal Resources Centre (Murdoch, Western Australia). All mice were maintained under standard specific pathogen free (SPF) housing conditions and all animal experiments were carried out according to the animal ethics guidelines endorsed by the National Health and Medical Research Council of Australia, and protocols approved by the University of Western Australia Animal Ethics Committee.

### Cell lines

The asbestos induced murine mesothelioma cell lines AB1 42, AB1‐HA 43, 44, and the gemcitabine resistant derivative AB1‐HA^GR^ 45; Renal cell carcinoma, (Renca) 46; 4T‐1 mammary carcinoma 47 and colon carcinoma cell lines CT26 (ATCC, CRL‐2947) 48 and CT44 49 have previously been described. All cells were maintained in RPMI 1640 (Life Technologies, Cat#11875) supplemented with 20 mM HEPES (Sigma–Aldrich, Cat#H3375), 0.05 mM 2‐ME (Sigma–Aldrich, Cat#M3148), 60 µg/ml penicillin (Sir Charles Gairdner Hospital Pharmacy, Perth), 50 µg/ml gentamicin (Life Technologies, Cat#15750078), 10% foetal calf serum (FBS; Life Technologies, Cat#12664‐025 lot#599957), and 400 µg/ml Geneticin (G418; Life Technologies, Cat#10131027). Trypsinised (Sigma–Aldrich, Cat#59430‐C) adherent cells were counted and viability assessed by trypan blue exclusion prior to use.

### Tumor inoculation, vaccination and diphtheria toxin treatment

Tumor cell lines (Tu) were resuspended in phosphate buffered saline (PBS) at 5 × 10^6^ cells/ml and 100 µl injected subcutaneously (s.c.) into the shaved, right hand flank of mice. Diphtheria toxin (DTX; Sigma–Aldrich, Cat#D0564) was prepared in sterile PBS and stored as 100 ng/μl single use aliquots at −80 °C until needed. Stocks of DTX were further diluted to 1–2 ng/μl and administered via intraperitoneal (i.p.) injection at indicated doses in two sequential injections one day apart (q1dx2). For vaccination experiments, mice received a s.c. injection at the base of the tail containing 100 μg CL4 peptide (IYSTVASSL, H2‐K^d^, Mimotopes), 50 μg PolyI:C (Invivogen, Cat#tlrl‐pic‐5) and 50 μg anti‐CD40 (Bioceros, clone FGK45) in a total volume of 200 μl PBS at indicated time points. tumor size was monitored by electronic callipers and calculated by multiplying the length and width to produce area in square millimeters (mm^2^). Mice were euthanized when s reached 150 mm^2^ according to approved UWA and Perkins Bioresources Animal Ethics protocols.

### Flow cytometry

Flow cytometry was performed using a BD Canto II or LSR Fortessa with at least 50,000 events were acquired for the lymphocyte gate. All antibodies are anti‐mouse unless otherwise stated. Single cell suspensions were prepared form peripheral blood (BL), tumor draining (dLN) or non‐draining lymph nodes (ndLN) and spleen (Spln) as previously described 50. Tumor samples were processed using the gentleMACS™ Octo Dissociator (Miltenyi Biotec Pty. Ltd, Cat#130095937) as per manufacturer instructions. Samples were stained using the following Treg Panel: αCD3 PE‐Cy7 (clone 145‐2C11, Cat#100320), αCD4 PerCP‐Cy5.5 (clone GK1.5, Cat#100434), and αCD278 (ICOS)‐APC (clone C398.4A, Cat#107706) all BioLegend; αCD8‐APC ef780 (clone 53–6.7, Cat#47‐0081‐82), αFoxP3‐FITC (clone FJK‐16s, Cat#11‐5773‐82) from eBioscience, and α‐Hu/mouse‐Ki67‐PE (clone B56, Cat#556027) and αCD25‐BV421 (clone PC61, Cat#562606) from BD Biosciences. Analysis was performed using Flow Jo (V10) with T cell subsets identified as CD8 (CD3+CD8+) cells, CD4 (CD4+FoxP3‐CD25‐/+), and Treg (CD4+FoxP3+CD25+).

### Statistics

Unpaired, nonparametric (Mann–Whitney) Student's t‐test was used to measure significance between two individual groups unless otherwise stated. Mixed model analysis of variance was used to analyse difference in growth. Log rank (Mantel–Cox) analysis was performed on survival curves. All analysis was performed using Graph Pad Prism Software (Graph Pad Software Inc., CA), or IBM^®^ SPSS^®^ statistics version 22 (Armonk, NY). A *P* value <0.05 was considered significant.

## Conflict of Interest

Dr Louis Boon is a shareholder from Cluster 4D Therapeutics BV that develops an anti‐human CD40 monoclonal antibody. All other authors declare no other commercial or financial conflict of interest.
